# Myalgic encephalomyelitis/chronic fatigue syndrome and encephalomyelitis disseminata/multiple sclerosis show remarkable levels of similarity in phenomenology and neuroimmune characteristics

**DOI:** 10.1186/1741-7015-11-205

**Published:** 2013-09-17

**Authors:** Gerwyn Morris, Michael Maes

**Affiliations:** 1 Tir Na Nog, Pembrey, Llanelli, UK; 2 Department of Psychiatry, Chulalongkorn University, Bangkok, Thailand; 3 Department of Psychiatry, Deakin University, Geelong, Australia

**Keywords:** Encephalomyelitis disseminata, Myalgic encephalomyelitis, Chronic fatigue syndrome, Inflammation, Autoimmunity, Oxidative and nitrosative stress, Mitochondria

## Abstract

**Background:**

‘Encephalomyelitis disseminata’ (multiple sclerosis) and myalgic encephalomyelitis/chronic fatigue syndrome (ME/CFS) are both classified as diseases of the central nervous system by the World Health Organization. This review aims to compare the phenomenological and neuroimmune characteristics of MS with those of ME/CFS.

**Discussion:**

There are remarkable phenomenological and neuroimmune overlaps between both disorders. Patients with ME/CFS and MS both experience severe levels of disabling fatigue and a worsening of symptoms following exercise and resort to energy conservation strategies in an attempt to meet the energy demands of day-to-day living. Debilitating autonomic symptoms, diminished cardiac responses to exercise, orthostatic intolerance and postural hypotension are experienced by patients with both illnesses. Both disorders show a relapsing-remitting or progressive course, while infections and psychosocial stress play a large part in worsening of fatigue symptoms. Activated immunoinflammatory, oxidative and nitrosative (O+NS) pathways and autoimmunity occur in both illnesses. The consequences of O+NS damage to self-epitopes is evidenced by the almost bewildering and almost identical array of autoantibodies formed against damaged epitopes seen in both illnesses. Mitochondrial dysfunctions, including lowered levels of ATP, decreased phosphocreatine synthesis and impaired oxidative phosphorylation, are heavily involved in the pathophysiology of both MS and ME/CFS. The findings produced by neuroimaging techniques are quite similar in both illnesses and show decreased cerebral blood flow, atrophy, gray matter reduction, white matter hyperintensities, increased cerebral lactate and choline signaling and lowered acetyl-aspartate levels.

**Summary:**

This review shows that there are neuroimmune similarities between MS and ME/CFS. This further substantiates the view that ME/CFS is a neuroimmune illness and that patients with MS are immunologically primed to develop symptoms of ME/CFS.

## Background

‘Encephalomyelitis disseminata’/multiple sclerosis (MS) and myalgic encephalomyelitis/chronic fatigue syndrome (ME/CFS) are both classified as diseases of the central nervous system by the World Health Organization (WHO). MS exhibits an almost bewildering radiological, clinical, and pathological heterogeneity. Evidence reveals that different processes, such as autoimmunity, inflammation and virus infection, may induce the pathology characteristic of the disease and suggests that MS is an illness involving the presence of different causative mechanisms. Distinct patterns of demyelination have been repeatedly documented. Two patterns bear a very close resemblance to autoimmune encephalomyelitis induced either by T cells alone or T and B cells in combination. The other patterns are highly indicative of virus infection or demyelination generated by exposure to environmental toxins rather overt autoimmune mechanisms. Pathological, biochemical and immunological data indicate that different pathways are generating the distinct pathology visible in different MS patients [1]. Research repeatedly demonstrates a proinflammatory milieu in MS patients as reflected by elevated levels of proinflammatory cytokines [2,3].

Patients with ME/CFS experience disabling levels of fatigue, as do people with MS, and they also have a wide range of neurological signs. The latter involve neurocognitive and autonomic symptoms, for example, postural hypotension and orthostatic intolerance [4,5] and a wide range of abnormalities on brain scans indicating elevated levels of lactate, cerebral hypoperfusion, and glucose hypometabolism [5]. Evidence shows that different trigger factors, such as infections and (auto)immune disorders, may be associated with the onset of ME/CFS [6]. ME/CFS patients display numerous immune abnormalities indicating an activated but dysregulated immune system, including chronically elevated levels of cytokines, signs of immune activation, loss of T cell homeostasis, decreased natural killer cell activity and autoimmune responses [6].

This review aims to compare the diseases of MS and ME/CFS on several different dimensions. These dimensions will include phenomenological similarities, including symptoms and course, elevated oxidative and nitrosative stress (O+NS), the existence of autoimmunity, cell mediated immunity and cytokine abnormalities and abnormalities in T cell activation and homeostasis, and a comparison of brain imaging findings. It is worth noting at the onset, however, that although CFS is recognized as an alternative term to myalgic encephalomyelitis there are many instances in the literature where the term is used as a synonym for fatigue of a psychiatric or idiopathic origin and patients are selected who only experience fatigue [7]. In this review we only consider data from studies where patients are recruited using CDC criteria and eschew studies where patients are selected because they meet arbitrary criteria produced by unvalidated or invalidated symptom questionnaires or generic fatigue scales. Trying to synthesize results where selection criteria are major confounding variable is virtually impossible, and in any event can lead to false conclusions [8].

## Discussion

### Phenomenological similarities between MS and ME/CFS

Many patients with MS have symptoms that are characteristic for ME/CFS. We will first discuss the typical symptoms of ME/CFS and then show that many patients with MS also have ME/CFS symptoms in conjunction with typical neurological deficits. People with ME/CFS can have a wide range of symptoms [9]. Typical symptoms include chronic fatigue, hyperalgesia, migraine-type headaches, unrefreshing sleep or even sleep/wake cycle reversal. Patients also present with symptoms consistent with a chronic influenza-like syndrome. These symptoms include unrelenting severe disabling fatigue in the physical and mental domains combined with incapacitating levels of muscle fatigability. Problems with memory retrieval and formation are also frequently observed. Problems with word retrieval mean that patients are frequently unable to finish sentences. These symptoms are all made worse by increases in cognitive and or physical activity. An inability to tolerate even trivial increases in physical or mental activity above individual norms is the hallmark symptom of ME/CFS [5]. This intolerance manifests itself in disease exacerbation, which may be short lived or prolonged [10,11]. ME/CFS patients display abnormalities in parameters appertaining to sympathetic and parasympathetic nervous system activity [12,13]. Orthostatic intolerance, and neurally mediated hypotension are commonly reported cardiovascular symptoms [4,14]. Postural orthostatic tachycardia syndrome (POTS) is another common finding. Exaggerated postural tachycardia and enhanced sympathetic activity have been reported [12,15]. Autonomic symptoms also include an intolerance of wide temperature fluctuations and grossly impaired thermostatic stability. A diminished cardiac response to exercise has also been demonstrated [16]. Resting sympathetic overactivity coupled with reduced vagal modulation appears to be a reproducible finding in ME/CFS [17]. De Becker *et al*. [18] reported a sympathetic drive increased heart rate (HR) on tilt compared to controls. Another study demonstrated impaired HR responses indicating the existence of attenuated cardiac sympathetic responsiveness. These lower HR responses could not be reconciled with an explanation based on patient deconditioning or prolonged inactivity [19]. Those authors reported that hemodynamic responses in patients with ME/CFS were significantly impaired during exercise compared to healthy controls. A number of other workers have reported autonomic dysregulation in people with ME/CFS [20-27]. Patients with ME/CFS typically use energy conservation strategies or pacing as a method of minimizing the effects of their fatigue on daily living [28].

Around 53% to 92% of MS patients experience disabling levels of fatigue [29]. Patients with MS frequently report that their fatigue is unremitting and relentless [30-32], and causes daytime sleepiness [33] and an irresistible urge to rest [32]. Clinically, fatigue presents as exhaustion, loss of energy, daytime somnolence, or exacerbation of symptoms. Activity usually acts to increase fatigue levels in MS [34]. Thus, the experience of permanent exhaustion is magnified by exacerbations leading to complete absence of energy after physical or cognitive activity [35,36]. Rapid exhaustion and markedly reduced exercise tolerance is a source of profound disability in patients with MS [36]. People with MS not only report a lack of energy, but also a profound intolerance of even minor physical activities [31,33,37]. MS patients often have concentration difficulties or an inability to complete mental tasks [30,33,37]. Reports of malaise are also commonplace [32,37].

The most frequent symptoms of autonomic dysfunction in MS patients are impotence, gastrointestinal dysfunction, sleep disturbances, disordered micturition and orthostatic intolerance [38,39]. Patients may develop POTS as a result of their underlying autonomic dysfunction [40]. A number of different groups have reported orthostatic dysregulation, neurocardiogenic syncope and cardiac dysrhythmias [41-44].

People with MS also use pacing strategies to minimize the effects of their fatigue on daily living. Such strategies include planning daily routines, ensuring that periods of higher activity take place in the mornings and budgeting time for rest or even sleep between such periods of increased activity [37]. A number of studies have empirically examined the effectiveness of pacing by enrolling patients in a course where they were given instructions in various pacing techniques [45-47]. These courses resulted in significant reductions in fatigue.

In summary (see Table 1), patients with ME/CFS and MS both experience severe levels of disabling fatigue and a worsening of symptoms following exercise and resort to energy conservation strategies in an attempt to meet the energy demands of day-to-day living. Debilitating autonomic symptoms are experienced by people with both illnesses. Diminished cardiac responses to exercise are a common finding as are reports of orthostatic intolerance and postural hypotension. It appears, however, that ME/CFS patients may be more sensitive to physical or cognitive activities than patients with MS.

**Table 1:** Phenomenological similarities between encephalomyelitis disseminata/multiple sclerosis (MS) and myalgic encephalomyelitis/chronic fatigue syndrome (ME/CFS)

**Phenomenology**	**MS**	**ME/CFS**
Disabling fatigue	**✓**	**✓**
Severe exercise intolerance	**✓**	**✓**
Mental fatigue	**✓**	**✓**
‘Pacing’ as an energy conservation strategy	**✓**	**✓**
Worsening of symptoms following exercise	**✓**	**✓**
Orthostatic intolerance	**✓**	**✓**
Gastrointestinal dysfunction	**✓**	**✓**
Cardiac dysrhythmias	**?**	**✓**
Postural hypotension	**✓**	**✓**
Diminished cardiac response to exercise	**✓**	**✓**
Relapsing-remitting nature	**✓**	**✓**
Chronic course	**✓**	**✓**
Disease exacerbated by infections	**✓**	**✓**
Disease exacerbated by psychological stress	**✓**	**✓**
Disease worsened or precipitated by infections	**✓**	**✓**

### Similarities in the course and other disease characteristics of MS and ME/CFS

There are four MS types, outlined below. (1) Patients with relapsing-remitting type MS endure relapses or episodes of acute impairment of neurologic function. Relapses may be replaced by times of partial or complete remissions without further exacerbations. This is easily the most common presentation of MS and people with relapsing-remitting MS make up about 85% of the MS population. (2) Patients with primary-progressive type MS endure a continuous deterioration of their disease typically, but not exclusively, without a pattern of relative relapse or remission. This phenotype is relatively rare comprising about 10% of the MS population. (3) Patients with secondary-progressive type MS endure an initial pattern of relapsing-remitting disease followed by a progressive deterioration of disease activity amidst a pattern of minor relapses and remissions. Around half of those with the relapsing-remitting course of disease will go on to develop secondary-progressive MS in the absence of treatment. (4) Patients with progressive-relapsing type MS experience a progressive worsening of symptoms from onset coupled with acute exacerbations with or without recovery. There is no period of remission in this form of MS. The frequency of this phenotype is approximately 5% of the total MS population.

In addition, ME/CFS is a chronic, relapsing-remitting disease involving the waxing and waning of symptoms [48-51]. The majority of studies examining longitudinal changes in disease activity using internationally agreed diagnostic criteria report a relapsing-remitting or progressive pattern of disease in the patients monitored [52-55]. Recovery from ME/CFS is extremely rare [53-55]. The average figure reported in studies using 1988 or 1994 Centers for Disease Control and Prevention recruitment criteria is some 4% [53,55,56]. Peterson *et al*. [57] reported a relative remission in some 40% of patients monitored over 12 months while 20% of patients demonstrated progressive worsening of their disease and 40% remained stable. Unfortunately, none recovered. Saltzstein *et al*. [58] reported very similar statistics.

Different trigger factors such as infections and other environmental factors play a role in the onset of MS and ME/CFS. Infections have been implicated in relapse of MS. Activation of immune pathways, mostly via cytokines, is believed to be responsible for the occurrence of severe relapses during or following infection [59]. Stress also increases the frequency of relapses or a general worsening of symptoms [60]. Stress leads to elevated cytokines and increases in general proinflammatory states, which may well create the environment fostering increased disease activity [61,62].

The vast majority of ME/CFS patients endure multiple persistent bacterial and viral infections [63-70]. The existence of these infections correlates positively with the total number of symptoms and the severity of those symptoms including the neurological symptoms [66]. Concurrent infections appear to worsen symptoms globally [71]. Stress is also related to worsening symptoms of ME/CFS [72,73].

The incidence and prevalence of MS demonstrates considerable geographic variability [74,75]. High frequency areas (prevalence of 60 per 100,000 or more) include Europe, the area encompassing the northern USA and Southern Canada, the antipodes including all of New Zealand, the south eastern part of Australia. In the US, the prevalence is 100 per 100,000. At a rate of 300/100,000, the residents of the Orkney Islands in the UK have a disproportionately heavy burden.

Estimating the prevalence of ME/CFS is a difficult exercise however. Although the disease was first reported in 1934, the introduction of new descriptive terminology in 1988 following an outbreak of ME in the USA led many physicians to equate the disease with idiopathic chronic fatigue. The presence of chronic fatigue in a population is common, ranging from just under 3,000 to just over 6,000 cases per 100,000 [76]. While disabling fatigue is certainly present in most people it is just one of an array of disabling neurological and neuroendocrine symptoms experienced by patients with ME/CFS. With that proviso in mind, the figures would indicate that some 0.2% of the people in the USA have ME/CFS [76-80], which is twice the prevalence of MS. As in many autoimmune disorders, ME/CFS and MS are more prevalent in women than in men [9,81].

Table 1 shows the phenomenological similarities between both disorders. Both MS and ME/CFS show a chronic and/or relapsing-remitting course, while infections and stress appear to play a large part in worsening of symptoms in both illnesses. Immune activation following infection is believed to trigger relapses in MS and ME/CFS. Patients with ME/CFS have more concomitant infections and the number of different infections correlates with the severity of symptoms. Stress is related to worsening of symptoms of fatigue in both MS and ME/CFS.

### Oxidative and nitrosative stress

Accumulating data demonstrates that O+NS plays a significant role in the pathophysiology of MS [82,83]. Studies have revealed the existence of lipid peroxidation in MS as evidenced by elevated levels of the alkanes pentane and ethane, hydrocarbons produced by peroxidation of unsaturated fatty acids [84]. O+NS likely make a major contribution to the pathophysiology of lesions in patients with MS [85]. Peroxinitrite for example is capable of modifying lipid, protein, DNA and mitochondrial structures and functions via the production of oxidizing and nitrating free radicals. Evidence supporting the presence of O+NS in demyelinating and inflammatory lesions includes the existence of nitrotyrosine together with lipid and protein peroxides [86,87]. Increased nitric oxide (NO) and inducible nitric oxide synthase (iNOS) production have been detected in peripheral blood mononuclear cells caused by raised levels of oxidative stress [87,88]. Isoprostanes, isomers of prostaglandins, are generated by peroxidation of fatty acids and can be detected in the urine as well as in the plasma from people with MS [89-91]. Elevated levels of isoprostane 8-epi-prostaglandin are found in the cerebrospinal fluid (CSF) of patients with MS [92].

Many studies using peripheral blood measures have shown increased O+NS in patients with ME/CFS, including increased levels of malondialdehyde (MDA), isoprostane, 8-OH-deoxyguanosine, 2,3 diphosphoglyceric acid, thiobutyric acid, and protein carbonyls [93-101]. The production of iNOS is significantly increased in ME/CFS patients as compared with normal controls [102]. As we will discuss below, there is also evidence that there is a chronic hyperproduction of NO [93]. Raised oxidative stress levels also occur in response to exercise in ME/CFS [103] potentially explaining one of the mechanisms underlying post-exertional malaise. In ME/CFS patients, exercise induces striking changes in the excitability of muscle membranes [104]. Skeletal muscle oxidative imbalance contributes to increased muscle fatigability [105]. Several authors have reported that O+NS measures demonstrate a significant and positive correlation with symptom severity [93-96,99,101,106,107].

Both MS and ME/CFS are accompanied by significantly depressed levels of crucial antioxidants and antioxidant enzymes. Syburra and Passi [108] reported low levels of vitamin E, ubiquinone (coenzyme Q10) and glutathione (GSH) peroxidase in MS. de Bustos *et al*. [109], however, did not find significant differences in serum levels of coenzyme Q10 between patients with MS and controls. There are reports on lowered levels of glutathione in the brains of MS patients [110]. Lowered zinc levels have been observed in MS [111].

Lowered levels of zinc, coenzyme Q10 and glutathione have been reported in ME/CFS [95,107,112]. A recent proton magnetic resonance spectroscopy study reported decreased cortical glutathione levels in the brain in ME/CFS that inversely correlated with lactate levels [107]. A study examining blood vitamin E levels showed that amelioration of oxidative stress occurs when ME/CFS patients enter remission [100].

Table 2 shows the similarities in O+NS pathways between both disorders. Markers of elevated O+NS are found in both illnesses. Moreover, the abnormalities reported are virtually identical in both illnesses and include elevated levels of peroxinitrite, NO and iNOS. Evidence of nitrosatively modified amino acids and proteins and oxidatively modified lipids are found in both illnesses. Reduced levels of antioxidants, including vitamin E, zinc and glutathione are also found in both diseases. While coenzyme Q10 is clearly related to fatigue in ME/CFS, the findings in MS are less evident.

**Table 2:** Similarities in oxidative and nitrosative stress (O+NS) pathways and antioxidant levels between encephalomyelitis disseminata/multiple sclerosis (ED/MS) and myalgic encephalomyelitis/chronic fatigue syndrome (ME/CFS)

**Oxidative and nitrosative stress (O+NS)**	**ED/MS**	**ME/CFS**
Lipid peroxidation	**✓**	**✓**
Increased malondialdehyde	**?**	**✓**
Elevated peroxynitrite	**✓**	**✓**
Nitrated amino acids	**✓**	**✓**
Elevated nitric oxide (NO)	**✓**	**✓**
Elevated inducible NO synthase (iNOS)	**✓**	**✓**
Raised isoprostane levels	**✓**	**✓**
Low vitamin E	**✓**	**✓**
Reduced levels of glutathione	**✓**	**✓**
Low zinc levels	**✓**	**✓**
Low coenzyme Q10 concentrations	**?**	**✓**
O+NS implicated in pathology	**✓**	**✓**

### Cytokine levels in MS and ME/CFS

Studies have repeatedly reported elevated levels of the proinflammatory cytokines, IL-1β [113-115], TNFα [115,116] and IL-6 [117,118] in the CSF and plasma of people with MS. Moreover, the cytokine pattern observed in the CSF of patients with relapsing-remitting MS varies depending on the stage of the disease [119]. Th1-like cytokines, such as IL-2, and interferon (IFN), and interleukin (IL)-12, are increased during active disease. T helper (Th)2 cytokines, such as the anti-inflammatory IL-10, transforming growth factor (TGF)-β and IL-4, are elevated during relative remission [120-123]. Th1 and Th2 cytokines are present in the CSF and lesions [124,125] during relapse and remission. The high concentrations of tumor necrosis factor (TNF)α and IL-10 (both in serum and CSF), which accompany an MS attack, suggest a concomitant expression of Th1 and Th2 cytokines and not to the sequential expression of Th1 cytokines followed by Th2 cytokines [126].

A number of studies have found increased levels of the major proinflammatory cytokines TNFα and IL-1β in ME/CFS (for a review see Maes *et al*. [127]). Recent evidence has challenged the view that patients with ME/CFS display an activated Th2 dominated immune system [5,128]. Proinflammatory and anti-inflammatory cytokines are known to coexist also in ME/CFS, although in many patients proinflammatory cytokines are dominant [127,129,130]. Studies examining the Th cytokine profiles in people with ME/CFS also show a large number of different findings almost certainly for methodological inconsistencies, including patient selection [5]. Rose *et al*. [131] reported that there was a significant upregulation of cyclo-oxygenase 2 (COX2), usually accompanied by increased iNOS, in MS lesions and opined that COX2 promoted excitotoxic death and damage of oligodendrocytes by coupling with iNOS. The involvement of COX2 in oligodendrocyte death was confirmed by Carslon *et al*. [132] using histopathological techniques. Upregulation of nuclear factor (NFκB in lesion-based macrophages amplifies the inflammatory reaction by stimulating the production of adhesion molecules and proinflammatory cytokines [133]. Activated NFκB is found at high levels in microglia of active lesions [134]. These authors proposed that high NFκB levels explains the relative rarity of oligodendrocyte death in MS. Generally it seems that upregulation of NFκB in neurons is protective but activation of NFκB in microglia stimulates neuronal degeneration [135,136]. Maes *et al*. [102] reported significantly elevated levels of COX2 and NFκB in patients with ME/CFS compared to healthy controls. Moreover, the severity of the illness correlated significantly and positively with the elevation in concentrations COX2 and NFκB.

Table 3 displays the similarities in immunoinflammatory pathways between MS and ME/CFS. Overall, proinflammatory cytokines are elevated in MS and ME/CFS but the results of investigative trials depend on methodology and can vary according to the state of the disease. Th1 and Th2 cytokines coexist in both illnesses. COX2 and NFκB are upregulated in both disorders and may play a role in the pathophysiology of both MS and ME/CFS.

**Table 3:** Similarities in immunoinflammatory pathways between encephalomyelitis disseminata/multiple sclerosis (ED/MS) and myalgic encephalomyelitis/chronic fatigue syndrome (ME/CFS)

**Immunoinflammatory pathways**	**ED/MS**	**ME/CFS**
Raised levels of proinflammatory cytokines, for example, interleukin (IL) 1 and tumor necrosis factor-α	**✓**	**✓**
Increased nuclear factor κB	**✓**	**✓**
Increased cyclo-oxygenase 2	**✓**	**✓**
Raised IL-2	**✓**	**✓**
Raised IL-10	**✓**	**✓**
Raised transforming growth factor β	**✓**	**✓**
Coexistence of a T helper (Th)1 and Th2 response	**✓**	**✓**
Elevated osteopontin levels	**✓**	**✓**
Temporal variation in cytokine profile	**✓**	**✓**
Elevated neopterin	**✓**	**✓**
T regulatory (Treg) dysfunction	**✓**	**✓**
Forkhead box P3 (FOXP3) dysfunction	**✓**	**✓**
Clonal exhaustion of T cells	**✓**	**✓**
Elevated CD26	**✓**	**✓**
CD69 expression	**↑**	**↓**
Low natural killer cell activity	**✓**	**✓**
Chronic activation of immunoinflammatory pathways	**✓**	**✓**

### Cell mediated immunity in MS and ME/CFS

#### Neopterin

Levels of neopterin have been reported as being higher in CSF of MS patients during exacerbations in comparison with remissions [136]. Increased urinary neopterin to creatinine ratio is an accurate surrogate marker of cell-mediated immune activation in MS [137]. Relapses and disease activity are related to increased neopterin levels [138-140]. Many studies have detected high levels of serum neopterin in ME/CFS [127,141-144].

#### Regulatory T cells

T cells are anergized in patients with MS in remission [145] indicating functioning regulatory T (Treg) cells. The situation in active disease is quite different however. Programmed death 1 (PD-1)-regulatory T cells are elevated in the peripheral blood of relapsing-remitting MS with active disease [146] and these Treg cells appear to be clonally exhausted [146,147] and only a small fraction express PD-1 receptors which are needed for their suppressive function [146]. An absence of PD-1 expression on forkhead box P3 (FOXP3) + CD4+ T cells greatly reduces their ability to suppress the activity of effector T cells, which is essential if self-tolerance is to be maintained and autoimmunity to be prevented [148]. A number of studies report impaired function of Tregs in active disease [149,150]. Tregs can react to inflammation by increasing numbers in active disease in an attempt to restore homeostasis [151]. PD-1 also has a key role in the modulation of T cell function during a prolonged viral infection. The functional impairment of T cells that occurs during chronic viral infections is considered to be due to T cell exhaustion promoted by activation of the PD-1 pathway and elevation of Tregs as the infection progresses [148,152,153]. Defective Treg function in people with MS is shown by the existence of Th17 lymphocytes in the peripheral circulation and the CNS [154-156]. Patients with relapsing-remitting MS display a Th1/Th17 phenotype in active disease [157,158]. Reduction in Treg function leads to a Th1/Th17 phenotype resulting from activation of naive T cells [159,160].

Compared to healthy controls, ME/CFS patients display statistically significant increases in CD4(+)CD25(+) Treg cells and FOXP3 expression [161]. Interestingly, chronically elevated IL-2 expression leads to the exhaustion of FOXP3 expression on CD25 + CD4+ Treg cells over time [159]. IL-2 is found chronically activated in MS [162-165] and ME/CFS [166,167]. This also indicates that a chronically activated immune system may exist in both diseases caused by a failure of the FOXP3/IL-2 feedback mechanism [168]. The pattern of raised IL-2 levels and chronic immune activation with disrupted homeostasis [169,170] coupled with raised levels of Treg cells seen in patients with ME/CFS strongly suggests Treg cell exhaustion as a feature of this disease as well. Strong evidence of T cell exhaustion has been reported in ME/CFS patients by many different researchers [171-175].

#### CD26+ and CD69+ T cells

Further evidence of an activated or dysregulated immune system is provided by a consideration of the data relating to the CD26 and CD69 T cell receptors in both MS and ME/CFS. CD26 is a T cell activation antigen with dipeptidyl peptidase 4 (DPPIV) activity [176]. CD26 as a surrogate marker of T cell activation correlates well with the activity of a number of autoimmune diseases [177,178]. CD26 is a marker of T cell activation and autoimmunity [179,180] and a key modulator of immune responsiveness [181]. CD26 expression is associated with Th-17 cells and IL-17 production is related to the CD26 + CD4+ T cell subset [182]. Memory CD4+ T cells with high expression of CD26+ correlate with clinical severity of MS [183,184]. The likelihood of a relapse is approximately three times higher in MS patients with high CD26 levels [185]. Importantly, elevated CD26 + CD4+ T cell numbers have been reported in people with ME/CFS compared to controls [186].

Another inhibitory regulator of Th17 cell differentiation is CD69, an early activation marker that promotes activation of the signal transducer and activator of transcription 5 (STAT5) pathway [187]. Patients with MS show an increased CD69 expression on T Lymphocytes [188]. The less pronounced IFN-induced effects on CD69 expression in MS versus controls is evidence of a defect in immunoregulation [189]. In ME/CFS, significantly lower CD69 expression on mitogen stimulated T cells has been detected [190]. This decreased expression of CD69 upon stimulation was strongly associated with inflammatory markers and indicates a defect in the initial activation of T lymphocytes and natural killer (NK) cells [112,190].

#### NK cells in MS and ME/CFS

Both MS and ME/CFS are accompanied by reduced and impaired activity of NK cells (NKCs). NKCs have a key role in immunoregulation. Crosstalk between NKCs and dendritic cells acts as a rheostat for the immune system and hence NKCs play a pivotal role in maintaining immune homeostasis [191] and in the suppression of T cell responses. In addition, NK CD56 bright cells have also a major role in combating autoimmunity. Benczur *et al*. [192] reported that NKC function was reduced in people with active disease compared with those in remission. Enhancement of NKC CD56 function leads to an amelioration of symptoms in MS [193]. Longitudinal disease activity, determined both clinically and by serial magnetic resonance imaging (MRI), correlates with natural killer cell activity (NKCA) and phenotype. Mean NKCA is significantly reduced in patients with MS as compared to normal controls. In relapsing-remitting MS, there is a significant association between lowered NKCA and the onset of new lesions on MRI [194]. In patients with MS, CD56 bright NKCs mediate immunoregulation [193]. Takahashi *et al*. [195] reported that a subset of NKCs was responsible for the maintenance of remission in relapsing-remitting MS and the development of relapses [192,195] and expansion corresponds with remission [196]. In ME/CFS, reduced and impaired NKC functioning has been a consistent finding reported by many authors [173,197,198].

It is tempting to speculate that gender-related differences in immune responsiveness may be associated with the higher prevalence of ME/CFS and MS in women as compared to men. The immunological processes occurring in the effector phase and induction of T cell priming are much stronger in female mice [81].

#### Summary

Table 3 shows that markers of immune activation are comparable in both illnesses. Elevated neopterin levels and elevated expression of the CD26 antigen on T cells demonstrate chronic immune activation while chronically elevated IL-2 levels indicate dysfunctional T cell activation and disordered homeostasis. Defective functionality of Treg cells is evidenced in both illnesses and clonal exhaustion of T cells is found in patients with MS and ME/CFS. There are, however, also differences between both disorders. Thus, clonal exhaustion may be limited to Treg cells in the active stage of MS, while the T cell exhaustion in patients with ME/CFS may be more global. While MS is characterized by increased in vivo expression of CD69, a decreased ex vivo CD69 expression is found in ME/CFS. Both MS and ME/CFS are accompanied by reduced NKCA.

### B cells and autoimmunity in MS and ME/CFS

#### B cells

Increased numbers of B cells are observed in ME and ME/CFS. CD80+ B cells numbers are increased in relapse phases of MS, relative to the values found in patients in remission or healthy controls [199]. Increases in the number of mature CD19 B cells have been reported in ME/CFS patients [200-202]. Klimas *et al*. [173] reported elevated numbers of CD20+ and CD21+ B cells in ME/CFS. Activated B cells possess a high capacity to generate inflammatory and regulatory cytokines and have a regulatory function in autoimmune diseases [203].

#### Autoimmunity

MS is widely considered to be at least in part an autoimmune disorder. Autoimmune reactions are also highly prevalent in ME/CFS. The presence of anti-nuclear antibodies and antibodies directed against cardiolipin and other phospholipids has been reported in some patients with MS [204-206]. Anti-neuronal antibodies, anti-muscle antibodies, anti-ganglioside antibodies [207-209] and anti-serotonin antibodies [210] are also active in MS.

Many individuals with ME/CFS show several indicators of autoimmune responses. Anti-cardiolipin antibodies have been reported in people with ME/CFS [211,212]. Konstantinov *et al*. [213] reported the presence of autoantibodies to nuclear envelope antigens. Anti-neuronal antibody levels are elevated in ME/CFS patients with neurologic abnormalities [214]. Other indicators include elevated antibody titers towards phospholipids, gangliosides and serotonin; anti-lamine SS DNA as well as anti-68/48 kDa and microtubule-associated proteone [215-217]. In addition to these increased antibody levels that are also observed in MS, patients with ME/CFS show various other markers of autoimmunity. Thus, autoantibodies against the muscarinic cholinergic receptor, mu-opioid receptor, 5-hydroxytryptamine (5-HT; serotonin) receptor 1A and dopamine receptor D2 have all been detected in ME/CFS patients [218] (for a review see [9]).

Both MS and ME/CFS are also accompanied by autoimmune reactions sometimes described as secondary. These reactions are directed against neoantigenic determinants (neoepitopes), which are created as a result of damage to lipids and proteins by O+NS [93,219]. Thus, the organisms may mount IgM and IgG mediated autoimmune reactions against oxidatively modified epitopes, such as fatty acids or byproducts of oxidative processes (for example, azelaic acid and malondialdehyde) and nitrosatively modified proteins [93]. Antibody titers against azelaic acid are higher during acute relapses in MS [220]. Anti-oleic acid conjugated antibodies have also been found in the sera of patients with MS when in acute relapse [221]. There is also a considerable amount of direct evidence of elevated protein S-nitrosation and nitrite content together with markedly increased levels of lipid peroxidation in the serum of patients with MS [222,223]. Specific IgM antibodies towards NO-modified amino acids and azelaic acid and malodialdehyde have been reported in MS patients [219]. The basic principle underpinning these data is that self-epitopes may be damaged by exposure to prolonged O+NS and thus lose their immunogenic tolerance and become a target for the hosts immune system.

The same IgM-related autoimmune responses can be detected in patients with ME/CFS, including autoimmune responses directed against disrupted lipid membrane components (palmitic, myristic and oleic acid), and residue molecules of lipid peroxidation, such as azelaic acid and malondialdehyde, IgM responses against the *S*-farnesyl-l-cysteine, and amino acids, modified by nitrating species such as nitrotyrosine, nitrophenylalanine, nitrotryptophan, nitroarginine and nitrocysteine, have all been reported [93,224]. These molecules have been damaged undergoing conformational change because of high levels of O+NS damage and have thus become immunogenic. The levels of these corrupted entities correlate positively and significantly with the severity of the ME/CFS symptoms [93].

#### Rituximab

The contribution of B cells to pathology in MS has been underlined by the evidence that rituximab, a monoclonal antibody that depletes CD20+ B cells in particular, has proven to be effective in the treatment of MS [225,226]. Rituximab has demonstrated efficacy in peripheral neurological diseases [227] by producing a sustained depression of pathogenic B cells [228]. In cerebrospinal fluid of MS patients, rituximab reduces not only B cells but also T cells [229]. This suggests that rituximab may well have properties other than as a monoclonal antibody to CD20+ B cells. T lymphocytes from relapsing-remitting MS patients demonstrate an impaired response to antigen stimulation following treatment with rituximab [230]. This finding supports the hypothesis that B cell activity is needed to maintain disease activity in MS [230,231]. The effectiveness of rituximab is not dependent on secreted antibody, as rituximab does not alter plasma cell frequencies in CSF or serum [232].

Rituximab has also some efficacy in the treatment of ME/CFS [233]. Thus, 30 patients with ME/CFS were randomized to rituximab or placebo in a placebo-controlled study and monitored for a calendar year. Positive responses were seen in 67% of the rituximab treated patients and 13% of the placebo group. The treatment improved symptoms globally. There were no serious side effects. The delayed nature of the responses (beginning from 2 to 7 months following rituximab treatment) lead the authors to conclude that ME/CFS was, at least in part, an autoimmune disease.

Rituximab is gathering momentum as a treatment in a variety of autoimmune diseases [234,235] especially where the patients are refractory to first and second line therapies [236]. When taken as a whole the trial data reveals that the vast majority of patients respond positively to rituximab. This benefit applies to people with rheumatoid arthritis [237,238], which is a licensed indication and other autoimmune conditions such as systemic lupus erythematosus [239] and Sjögren’s syndrome [240] where the drug is used off license. Rituximab appears to have a number of additional benefits in addition to CD20 B cell depletion which all act to normalize immune homeostasis. Rituximab has several effects on the immune system. One of the most potentially surprising effects is the reduction of Th17 T cell production [241,242]. These T cells are the cause of T cell induced autoimmunity and neurotoxicity in autoimmune diseases such as MS. Rituximab has a direct effect on reducing IL-2 levels and thus potentially inactivating a chronically activated immune system [243]. Rituximab achieves this by inhibiting the production of NFκB [244,245], which is another key mediator of autoimmunity. Rituximab also raises the function of Treg cells [246,247].

#### Summary

Table 4 displays the similarities in B cells and autoimmune responses between MS and ME/CFS. In all, the range of autoantibodies produced in both illnesses is vast and once again virtually identical. Antibodies are found against nuclear and neuronal antigens, cardiolipin, phospholipids, serotonin, and gangliosides. IgM responses are detected against oleic, palmitic and myristic acid in patients with ME/CFS, and oleic and palmitic acid in patients with MS. Antibodies towards the byproducts of lipid peroxidation, that is, azelaic acid and MDA, and *S*-farnesyl-l-cysteine, are found in patients with ME/CFS but only towards azelaic acid and MDA in patients with MS. Autoantibodies to nitrotyrosine are found in both illnesses but a wider range of autoantibodies to other nitrated amino acids are found in ME/CFS which have not been reported in MS. This hints at the fact that O+NS-induced autoimmune responses may be higher in patients with ME/CFS than in those with MS.

**Table 4:** Similarities in autoimmune responses between encephalomyelitis disseminata/multiple sclerosis (ED/MS) and myalgic encephalomyelitis/chronic fatigue syndrome (ME/CFS)

**Autoimmune responses**	**ED/MS**	**ME/CFS**
Increased numbers of B cells	**✓**	**✓**
Anti-nuclear antibodies	**✓**	**✓**
Anti-cardiolipin antibodies	**✓**	**✓**
Anti-phospholipid antibodies	**✓**	**✓**
Anti-neuronal antibodies	**✓**	**✓**
Anti-muscle antibodies	**✓**	**✓**
Anti-ganglioside antibodies	**✓**	**✓**
Anti-serotonin (5-hydroxytryptamine (5-HT)) antibodies	**✓**	**✓**
Anti-muscarinic cholinergic receptor antibodies	**-**	**✓**
Anti-mu-opioid antibodies	**-**	**✓**
Anti-5-HTA receptor antibodies	**-**	**✓**
Anti-D2 receptor antibodies	**-**	**✓**
IgM against oxidatively modified neoepitopes, for example, malondialdehyde, oleic and myristic and palmitic acid	**✓**	**✓**
IgM against nitrosatively modified neoepitopes, for example, NO adducts	**✓**	**✓**
Response to rituximab	**✓**	**✓**

### Mitochondrial dysfunctions in MS and ME/CFS

Many studies report changes in the levels of circulating compounds related to impaired energy metabolism, elevated O+NS, and impaired antioxidant status occurring in MS [248]. Biochemical abnormalities in levels of pyrimidines, creatine, malondialdehyde ascorbic acid, nitrate and nitrite strongly suggest a profound alteration in redox balance and energy metabolism in patients with MS [248,249]. Elevated levels of oxypurines in the serum are a product of abnormal purine nucleotide metabolism when adenosine triphosphate (ATP) production is insufficient to meet cellular demand [250,251]. Nuclear magnetic resonance imaging (NMRI) techniques allow the direct measurement of mitochondrial functioning, rate of glycolysis and availability of energy, and this approach is being increasingly used in MS research. For example, Lazzarino *et al*. [252] reported a significant degree of central ATP depletion in their MS patients and concluded that an increased energy demand and mitochondrial failure was the cause of that depletion. Mitochondrial damage may be driven by free radicals produced by activated microglia [253]. Damage to mitochondria and the ensuing energy failure are well known drivers for tissue injury [254]. In MS lesions, conformational changes are observed in proteins of the mitochondrial respiratory chain [255,256]. Deletions in mitochondrial DNA may be observed in neurons [257]. Mitochondrial DNA and proteins are both very vulnerable to damage by O+NS [258]. Therefore, it is likely that O+NS drive injuries to mitochondria and mitochondrial DNA in patients with MS [254,259,260].

The evidence that mitochondrial dysfunction and abnormally high lactate levels play a pivotal role in the pathophysiology of ME/CFS is expansive and expanding [261-264]. Vermeulen *et al*. [265] reported that in two exercise tests held 24 h apart, patients with ME/CFS reached their anaerobic threshold at a significantly lower oxygen consumption than healthy controls in the first test. This finding also applied to their maximal exercise capacity, which was also attained at a much lower oxygen capacity than the control group. This difference was even greater on the subsequent test. The researchers concluded that these findings demonstrated an increase in lactate production and decrease in ATP production relative to controls. Arnold *et al*. [266] using 31P nuclear magnetic resonance spectroscopy, revealed an abnormal increase in the level of intracellular lactic acid in the exercised forearm of a ME/CFS patient. This was proportional to concomitant changes in high-energy phosphates. Behan [261] reported finding structural mitochondrial abnormalities in the skeletal muscle of ME/CFS patients. ME/CFS patients display a significant increase in intracellular lactate levels following exercise compared to controls [263,267]. They display a significantly lower ATP resynthesis rate during recovery from exercise than normal controls stemming from impaired oxidative phosphorylation [263]. ME/CFS patients display an abnormal rise in lactate with even minor exercise and an extremely slow recovery from this state [262,263,265]. Fatigue can result from an accumulation of reactive oxygen species (ROS) and depletion of available ATP in muscle cells [268]. Patients with ME/CFS reach exhaustion at a much earlier time point than healthy controls. Upon the point of exhaustion, ME/CFS patients also have reduced intracellular levels of ATP indicating a defect of oxidative metabolism combined with an acceleration of glycolysis in the working skeletal muscles [269]. ME/CFS is accompanied by significantly increased ventricular lactate, indicating mitochondrial dysfunctions in the illness [107,270,271].

Table 5 displays the similarities in mitochondral dysfunctions between MS and ME/CFS. In all, there is considerable evidence that mitochondrial dysfunction is heavily involved in the pathophysiology of both MS and ME/CFS. Production of ATP is suboptimal and levels are depleted in the brain and/or striated muscles. Decreased phosphocreatine synthesis rates following exercise is indicative of abnormal metabolic responses to exercise in MS and ME/CFS. Impaired oxidative phosphorylation is an issue in both disorders but accelerated glycolysis in muscles has been reported in people with ME/CFS but not in people with MS. Conversely, damage to the mitochondrial respiratory chain in neurons has been reported in MS but not in ME/CFS.

**Table 5:** Similarities in mitochondral and brain dysfunctions between encephalomyelitis disseminata/multiple sclerosis (ED/MS) and myalgic encephalomyelitis/chronic fatigue syndrome (ME/CFS)

**Mitochondrial dysfunctions**	**ED/MS**	**ME/CFS**
Depleted ATP production (muscle and brain)	**✓**	**✓**
Decreased phosphocreatine resynthesis following exercise	**✓**	**✓**
Impaired oxidative phosphorylation	**✓**	**✓**
Acceleration of glycolysis	**?**	**✓**
Damage to mitochondrial respiratory chain in neurons	**✓**	**?**
Oxidative mitochondrial damage	**✓**	**✓**
Mitochondrial energy failure	**✓**	**✓**
Brain dysfunctions:		
Cerebral hypoperfusion	**✓**	**✓**
Reduced cerebral glucose metabolism	**✓**	**✓**
Gray matter atrophy	**✓**	**✓**
Increased cerebral lactate	**✓**	**✓**
Increased cerebral choline	**✓**	**✓**
Reduced levels of *N*-acetyl aspartate	**✓**	**✓**

### Brain dysfunctions

Single photon emission computed tomography (SPECT), used to examine cerebral perfusion in MS patients, showed significant decreases in blood flow in areas of cortical gray and white matter [272,273]. Cerebral hypoperfusion in MS patients relative to controls is evident in both the cortex and deep gray matter, and is especially pronounced in the thalamus and caudate nuclei. Generalized reduction in cerebral oxygen utilization and blood flow in white and gray matter correlates with cognitive impairment [274]. Raschid *et al*. [275] demonstrated reduced perfusion, particularly in the gray matter of MS patients belonging to the primary and secondary-progressive subgroups. The reduction was observed in both deep gray and cortical matter and suggests depressed neuronal metabolic activity or actual neuronal loss [275].

Patients with ME/CFS display a global reduction of brain perfusion, with a characteristic pattern of hypoperfusion in the brainstem [276,277]. SPECT abnormalities occur significantly more frequently and in greater numbers than MRI abnormalities do in patients with ME/CFS [278]. Using SPECT, a reduced cerebral blood flow was observed in the brain in 80% of ME/CFS patients [279]. Significant brain stem hypoperfusion has also been revealed in ME/CFS patients compared to healthy controls [276,280]. Fischler *et al*. [277] demonstrated a positive and significant association between neurocognitive impairments experienced by patients and reduced frontal blood flow.

Positron emission tomography (PET), using a labeled native glucose analogue, that is, [18F]fludeoxyglucose (FDG-PET), has revealed a positive correlation between clinical progression of MS and cerebral glucose metabolism [281,282]. Paulasu *et al*. [283] reported lowered glucose metabolism in the basal ganglia and frontal cortex of MS patients reporting severe levels of fatigue. The use of FDG PET in ME/CFS has revealed glucose hypometabolism in various areas of the brain [280,284].

Traditionally 1.5 T-weighted, T1-weighted and T2-weighted, non-contrast or gadolinium, and enhanced T1-weighted hyperintense MRI images have been used to monitor disease progress in the white matter of MS patients [285]. Both gray matter atrophy [285,286] and lesions [287,288] have also been revealed in the cerebral cortex and deep gray matter structures using MRI. Atrophy in the brains of people with MS is related to gray matter hypointensity [289]. However, sensitivity of the conventional MRI methods for gray matter lesions is low compared to white matter lesions [288,290]. MRI techniques are not sufficiently sensitive to detect purely cortical MS lesions [291]. This sensitivity can be improved using higher field strength [292,293] or voxel-based morphometry [294,295].

MRI involving voxel-based morphometry in patients with ME/CFS has revealed gray matter volume reduction [296-298]. These reductions are apparently unrelated to the duration of illness or the age of the person examined. Subcortical white matter hyperintensities have been repeatedly recorded in ME/CFS [299,300].

The use of proton magnetic resonance spectroscopy (MRS) has revealed abnormally high levels of cerebral lactate in patients with MS [301,302]. Using choline MRS, abnormally high choline was detected in the basal ganglia of patients [303-305]. Richards [306] using MRS demonstrated elevated concentrations of choline, lactate and lipids. In another study, proton MRS revealed significantly lower *N*-acetyl aspartate levels in the hippocampal areas of MS patients [307]. MS patients with active disease have high levels of lactate levels in CSF. This elevation in lactate levels may result from anaerobic glycolysis in activated leukocytes during active disease [308].

Brooks *et al*. [309] examined a cohort of ME/CFS patients using MRI and nuclear MRS. Using proton MRS, significantly reduced *N*-acetyl aspartate levels were observed in the hippocampal areas of ME/CFS patients. Chaudhuri *et al*. [310], using the same technique, demonstrated increased choline signaling in the basal ganglia of ME/CFS patients. The choline peaks in the basal ganglia most likely are related to ‘increased cell membrane turnover due reparative gliosis’ [310]. In addition, Puri *et al*. [311] established a significant choline/creatine signal in the occipital cortex of patients with ME/CFS. In children with ME/CFS, significant increases in the choline/creatine ratio as measured by MRS were observed [312].

Table 5 shows the similarities in brain dysfunctions between MS and ME/CFS. In summary, using SPECT, PET, and MRI, it was found that both disorders display cerebral hypoperfusion, reduced cerebral glucose metabolism and gray matter atrophy. Nuclear MRS has revealed abnormal choline signaling in the basal ganglia in both diseases coupled with elevated levels of lactate and reduced concentrations of *N*-acetyl aspartate.

### Mechanistic explanations of typical ME/CFS symptoms in MS and ME/CFS

**Figure 1:**
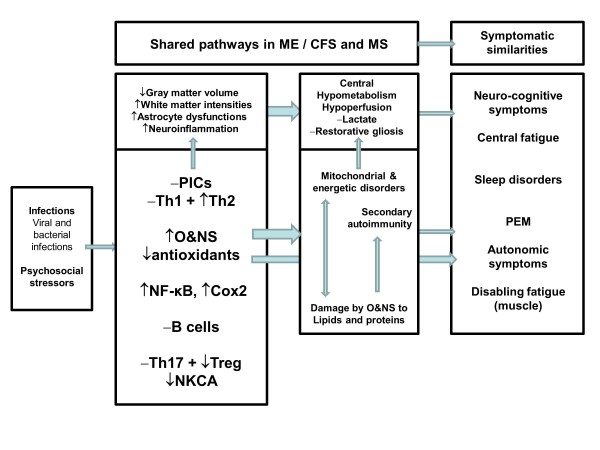
**Diagram integrating immune signaling and metabolic pathways, which together explain the symptomatic similarities between both multiple sclerosis (MS) and myalgic encephalomyelitis/chronic fatigue syndrome (ME/CFS).** Shared pathways are dysfunctions in intracellular signaling pathways, for example, nuclear factor κB (NFκB); immunoinflammatory pathways, for example, T helper (Th) and T regulatory (Treg) cells, cyclo-oxygenase 2 (COX2), and proinflammatory cytokines (PICs); and oxidative and nitrosative stress (O+NS) pathways. These in turn may induce increased damage by O+NS to proteins and lipids, secondary autoimmune responses and mitochondrial defects. There is evidence that these dysfunctions together with brain disorders are associated with the onset of ME/CFS symptoms, which appear in ME/CFS and MS. PEM = post-exertional malaise.

Above, we have already discussed that many patients with MS have typical ME/CFS symptoms, including fatigue and post-exertional malaise. In this section we will discuss the mechanistic explanations of the typical symptoms of ME/CFS that may occur in patients in MS. Fatigue in MS has both central (perception) and peripheral (impaired metabolism) components [313,314]. Figure 1 shows a diagram that integrates the numerous pathways into a mechanistic model emphasizing the shared and interactive immune signaling and metabolic pathways that explain the symptomatic similarities in both diseases.

A number of authors have suggested a role for the immunological abnormalities seen in people with MS in the production of the severe fatigue endured by so many patients. Flachenecker *et al*. [315] found that TNFα levels were significantly higher in people with fatigue than those without fatigue. Further support for this concept is found in studies that posit a mediative role for IL-6 as well as TNFα in the generation of fatigue in MS [316,317]. Pokryszko-Dragan *et al*. [318] reported that the severity of fatigue experienced by MS patients is significantly correlated to the stimulated production of IFNγ by T lymphocytes. Increased levels of proinflammatory cytokines are likely involved in the development and maintenance of fatigue in MS [319]. Raised levels of O+NS and mitochondrial dysfunctions could also conspire together to cause fatigue and the post-exertional malaise experienced by people with MS [8,128].

MS patients demonstrate an exaggerated metabolic response to exercise compared to controls, and thus metabolism appears to be a major contributing factor in creating the excessive muscle fatigue experienced by people with MS [320]. Patients with MS display objective and clinically significant levels of impaired functional capacity indicated by lower maximal oxygen consumption and maximal workload compared to sedentary controls. These objective abnormalities correlate positively and significantly with measures of fatigue [321]. Fatigability of striated muscle not related to central nervous system activity is a frequent manifestation of MS [322]. In addition, it has been shown that individuals with MS have a markedly lowered rate of phosphocreatine resynthesis after depletion compared with controls [323]. The reduced phosphocreatine resynthesis results from impaired oxidative ATP production, probably stemming from impaired oxidative enzyme activities [324]. Muscle in MS patients is significantly smaller than that found in healthy individuals and relies more on anaerobic than aerobic respiration [324]. Several studies have found abnormalities relating to defects in maximal voluntary contraction during exercise or after during the facilitation period in MS patients with muscle weakness [325] and the magnitude of the defects correlates with the degree of nerve damage [326]. Motor evoked potentials (MEPs) in MS tend to be abnormal in MS if people have disabling fatigue [327,328]. Impaired motor performance has been demonstrated in people with MS [329]. Ng *et al*. [330] reported that maximal voluntary contraction was 27% lower in MS patients than in the control group. The motor changes however were not related to fatigue but impaired walking ability. It is worthy of note that MS patients with a modest level of disability display gross reductions in exercise capability [331]. Savci *et al*. [332] noted that weakness in respiratory muscles, impaired lung function and degree of neurological impairment are not factors contributing to lowered functional exercise capability in MS patients.

Functional brain imaging research using SPECT and PET indicate that MS fatigue is connected to global glucose hypometabolism in the prefrontal cortex and the basal ganglia [282,333-336]. Other researchers [337,338] demonstrated that hypoperfusion correlates with disease and fatigue severity in MS. PET studies show that hypometabolism of particular brain areas, especially the frontal and subcortical circuits, is associated with fatigue [37,339,340]. MRI, PET and functional MRI studies indicate that fatigue is related to gray matter disease, in the thalamus and caudate areas and particularly the cerebral cortex [341].

In addition, the fatigue, fatigability and post-exertional malaise in ME/CFS have central and peripheral components [5,128]. As explained elsewhere and above, fatigue in ME/CFS is associated with and may be explained by increased levels of proinflammatory cytokines, O+NS and mitochondrial defects [5,128]. Several studies have found abnormalities relating to defects in maximal voluntary contraction during exercise or after during the post exercise facilitation period in ME/CFS [342]. MEP immediately following a period of exercise was significantly lower in ME/CFS and MEP facilitation 30 minutes after exercise was significantly less than in controls [343,344]. These parameters were also low in another study [345]. Some authors have proposed the hypothesis that the fatigue in ME/CFS is entirely of neurological origin [346,347]. This would however seem to be a minority viewpoint at this time.

Schillings *et al*. [348] reported impaired central activation in ME/CFS during maximal voluntary contraction and reported similar findings in seven out of the nine studies reviewed. Kent-Braun *et al*. [349] also reported markedly diminished levels of central activation at the end of a muscle contraction period. Schillings *et al*. [348] reported that the apparent central activation failure at maximal voluntary contraction in ME/CFS is of a similar magnitude to that reported in stroke and Amyotrophic Lateral Sclerosis [350,351]. A large number of studies demonstrate impaired motor performance in people with ME/CFS [345,352,353].

In patients with ME/CFS, Barnden *et al*. [297] reported that the volume of white matter, as measured using 3 T MRI, correlates significantly and positively with the severity of fatigue experienced by the patients. The authors noted hemodynamic abnormalities in the brainstem, deep frontal white matter, the caudal basal pons and hypothalamus, suggestive of impaired cerebrovascular autoregulation. When taken as a whole, the evidence pointed to astrocyte dysfunction and resetting of homeostatic norms. Astrocyte activity regulates cerebrovascular autoregulation [297] and cerebral blood flow [354,355]. Astrocyte malfunction is an important cause of mental fatigue [355]. Thus, dysfunctional astrocyte activity reported in ME/CFS would be expected to lead in a breakdown of mechanisms controlling blood flow in the brain. Patients with ME/CFS display a global reduction of brain perfusion, with a characteristic pattern of brainstem hypoperfusion [276,356]. The severity of disabling fatigue experienced by patients with ME/CFS is associated with the reduction in basal ganglia activation [357]. When taken as a whole, the evidence of gray matter abnormalities and astrocyte dysfunction as contributors to the fatigue experienced by those with both illnesses appears substantive.

In summary, fatigue and post-exertional malaise in MS and ME/CFS may be explained by peripheral and central mechanisms, including increased levels of proinflammatory cytokines, O+NS and mitochondrial dysfunctions. In both disorders, abnormalities relating to defects in maximal voluntary contraction during exercise are detected. Central disorders, including glucose hypometabolism and cerebral hypoperfusion, may contribute to fatigue in both disorders.

## Summary

MS and ME/CFS show remarkable levels of similarity in many dimensions. The ever-present disabling fatigue is a burden for both groups of patients to carry. This burden is made heavier by severe levels of exercise intolerance, which induce a worsening of symptoms in people with both illnesses. Both sets of patients resort to ‘pacing’ as an energy conservation strategy in an attempt to meet the energy demands associated with normal living. ME/CFS and MS are more prevalent in women than in men and show a chronic or waxing and waning course. Increased levels of O+NS occur in both illnesses and patients share an almost identical range of empirically determined abnormalities as evidenced by elevated levels of peroxinitrite, NO and iNOS, lipid peroxidation and nitration of amino acids. The consequences of O+NS damage to self-epitopes is evidenced by the almost bewildering and almost identical array of autoantibodies formed against damaged epitopes seen in both illnesses. Reduced levels of antioxidants, including vitamin E, zinc, glutathione are also found in both diseases. Evidence of chronic immune activation coupled with disordered T cell homeostasis is seen in both diseases. Similar abnormalities exist in levels of proinflammatory cytokines, serum neopterin and T cell antigens. Although reduced NKC function in ME/CFS has been emphasized over many years the identical abnormalities in MS has not received such widespread attention. The findings produced by neuroimaging using PET, SPECT and nuclear MRS are similar in both illnesses and in MS the severity of the abnormality in glucose metabolism correlates well with disease activity while MRI findings do not.

There are however also differences in symptomatic and immune profiles between both diagnoses. Thus, patients with ME/CFS seem more sensitive to increases in physical or cognitive activity than patients with MS. ME/CFS patients may have a worse experience with regard to infections, while the number of infections is associated with increasing symptom severity. While MS is characterized by increased expression of CD69, a decreased CD69 expression is seen in ME/CFS. Accelerated glycolysis is reported in ME/CFS but not in MS. Neuronal damage to the respiratory chain has been found in MS but not in ME/CFS. T cell exhaustion seems to be more of an issue in ME/CFS than in MS. When taken together the range of surrogate markers for O+NS and the range of autoantibodies are wider in ME/CFS than in MS and this may be due to an increased severity of O+NS in ME/CFS. While coenzyme Q10 is related to fatigue in ME/CFS, the findings in MS are less evident. Finally, while both ME/CFS and MS are chronic immunoinflammatory diseases, inflammation of the central nervous system is clearly more prominent in MS than in ME/CFS.

Nevertheless, the strong similarities between both disorders in terms of phenomenological, neurobehavior and neuroimmune characteristics further underscore that ME/CFS belongs to the spectrum of neuroimmune disorders. In addition, the data show that the comorbidity between both disorders and the high prevalence of ME/CFS symptoms in patients with MS may be explained by neuroimmune mechanisms.

**Figure 2:**
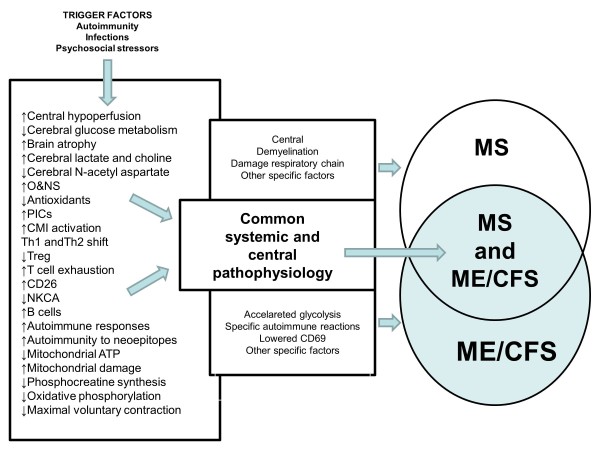
**The significant neuroimmune overlaps in multiple sclerosis (MS) and myalgic encephalomyelitis/chronic fatigue syndrome (MS/CFS) may be based on shared immunoinflammatory, oxidative and nitrosative stress (O+NS), autoimmune and mitochondrial pathways, and brain dysfunctions.** CMI = cell-mediated immunity; NKCA: natural killer cell activity; PICs = proinflammatory cytokines; Th = T helper; Treg = T regulatory.

Figure 2 shows that the significant comorbidity between MS and ME/CFS may be based on shared immunoinflammatory, O+NS, autoimmune and mitochondrial pathways and brain dysfunctions.

**Figure 3:**
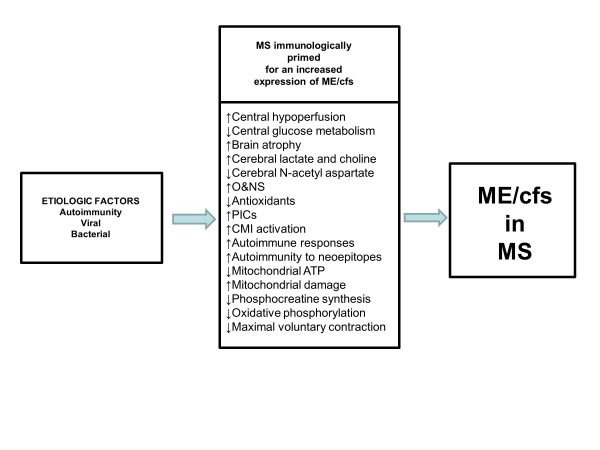
**A second model that explains the high incidence of characteristic symptoms of myalgic encephalomyelitis/chronic fatigue syndrome (MS/CFS) in multiple sclerosis (MS).** This model shows that MS patients are neuroimmunologically primed towards a higher expression of ME/CFS symptoms. Thus, the activation of immunoinflammatory, oxidative and nitrosative (O+NS), autoimmune and mitochondrial pathways, and brain dysfunctions may prime MS patients for an increased prevalence of ME/CFS symptoms. CMI = cell-mediated immunity; PICs = proinflammatory cytokines.

Figure 3 shows a second model that explains the high incidence of typical ME/CFS symptoms in patients with MS. This model suggests that patients with MS are neurologically and immunologically primed for an increased expression of ME/CFS symptoms. Thus, the activation of immunoinflammatory, autoimmune, mitochondrial and O+NS pathways together with brain disorders may prime MS patients for an increased prevalence of ME/CFS symptoms. Other possibilities are that ME/CFS could increase the odds to develop MS or when comorbid with MS could aggravate the severity of MS. It is also possible that there is a junction in immunoinflammatory progression that could explain bifurcation to ME/CFS rather than MS. For example, the initial lesions in ME/CFS could be smaller than in MS but at the expense of greater bioenergetic impairments [5].

## Abbreviations

COX2: Cyclo-oxygenase 2; CSF: Cerebrospinal fluid; DPPIV: Dipeptidyl peptidase 4; FDG: Fluodeoxyglucose; HR: Heart rate; IFN: Interferon; IL: Interleukin; iNOS: Inducible nitric oxide synthase; ME/CFS: Myalgic encephalomyelitis/chronic fatigue syndrome; MEP: Motor evoked potentials; MRI: Magnetic resonance imaging; MRS: Magnetic resonance spectroscopy; MS: Multiple sclerosis; NFκB: Nuclear factor κB; NKCA: Natural killer cell activity; NMRI: Nuclear magnetic resonance imaging; NO: Nitric oxide; O+NS: Oxidative and nitrosative stress; PD: Programmed death; PET: Positron emission tomography; POTS: Postural orthostatic tachycardia syndrome; ROS: Reactive oxygen species; SPECT: Single photon emission computed tomography; TGF: Transforming growth factor; Th: T helper; TNF: Tumor necrosis factor; Treg: T regulatory.

## Competing interests

No specific funding was obtained for this review. The authors declare that they have no competing interests.

## Authors’ contributions

GM and MM participated in the design of this review and contributed equally to this paper. All authors read and approved the final version.
